# Challenges in the Development of Drugs for Sarcopenia and Frailty - Report from the International Conference on Frailty and Sarcopenia Research (ICFSR) Task Force

**DOI:** 10.14283/jfa.2022.30

**Published:** 2022-04-01

**Authors:** Matteo Cesari, R. Bernabei, B. Vellas, R. A. Fielding, D. Rooks, D. Azzolino, J. Mariani, A. A. Oliva, S. Bhasin, Y. Rolland, ICFSR Task Force members

**Affiliations:** 1 Geriatric Unit, IRCCS Istituti Clinici Scientifici Maugeri, University of Milan (UNIMI), Via Camaldoli 64, 20138 Milano, Italy; 2 Department of Aging, Neurology, Orthopedic della Testa-Collo, Agostino Gemelli Policlinic Foundation, Roma, Italy; 3 Gérontopôle, CHU Toulouse, Toulouse, France; 4 Nutrition, Exercise Physiology, and Sarcopenia Laboratory, Jean Mayer USDA Human Nutrition Research Center on Aging at Tufts University, Boston, MA USA; 5 Translational Medicine, Novartis Institutes for Biomedical Research Inc., Cambridge, USA; 6 Biophytis, Université Pierre et Marie Curie, Paris, France; 7 Longeveron Inc., Miami, USA; 8 Boston Claude D. Pepper Older Americans Independence Center, Brigham and Women’s Hospital, Harvard Medical School, Boston, USA

**Keywords:** Aging, pharmacological interventions, physical performance, clinical trial, sarcopenia, frailty

## Abstract

Sarcopenia and frailty represent two burdensome conditions, contributing to a broad spectrum of adverse outcomes. The International Conference on Frailty and Sarcopenia Research (ICFSR) Task Force met virtually in September 2021 to discuss the challenges in the development of drugs for sarcopenia and frailty. Lifestyle interventions are the current mainstay of treatment options in the prevention and management of both conditions. However, pharmacological agents are needed for people who do not respond to lifestyle modifications, for those who are unable to adhere, or for whom such interventions are inaccessible/unfeasible. Preliminary results of ongoing trials were presented and discussed. Several pharmacological candidates are currently under clinical evaluation with promising early results, but none have been approved for either frailty or sarcopenia. The COVID-19 pandemic has reshaped how clinical trials are conducted, in particular by enhancing the usefulness of remote technologies and assessments/interventions.

## Introduction

**S**arcopenia and frailty are two major age-related conditions. Sarcopenia, first described by Irwin Rosenberg in 1988 (1), is defined as the loss of muscle mass and strength leading to poor physical function (2). On the other hand, frailty is a medical condition characterized by a multi-system impairment responsible for an increased vulnerability to endogenous and exogenous stressors (3).

Multiple pathophysiological mechanisms underlie the onset and manifestation of the two conditions. In particular, immunosenescence (i.e., the age-related dysfunction of the immune system) and inflammaging (i.e., the age-related low-grade chronic inflammatory state) are indicated as key determinants (4). Furthermore, sarcopenia and frailty synergistically contribute to the physical impairment of the older individual (5).

Significant research efforts have contributed to our understanding of frailty and sarcopenia. The two conditions have been frequently explored as simultaneously acting, and physical function impairment is often a common feature of both conditions. To date, no pharmacological treatment has yet been approved for either frailty or sarcopenia. Recommendations for the prevention and treatment of frailty and sarcopenia are thus still mainly based on lifestyle interventions, such as nutrition and physical exercise.

The lack of treatment options stems in part from the paradigm of standalone/single diseases traditionally adopted in medicine. This paradigm is inadequate for many older people, who often present with high level of clinical complexity due to the simultaneous presence of multiple interacting chronic conditions that often exist in syndromic constellations. Unfortunately, this suboptimal clinical paradigm of addressing single disease condition has also been largely mirrored in the process of drug development for older persons (6). Indeed, the complexity of age-related conditions (as sarcopenia and frailty) makes particularly challenging the study of pharmacological and non-pharmacological interventions in older persons. Geriatric research is indeed characterized by the need to consider many confounders and mediators potentially influencing the study findings, their interpretation, and generalizability to real life.

In 2012, the first International Conference on Frailty and Sarcopenia Research (ICFSR) took place in Toulouse, France. In parallel with the conference, an ICFSR Task Force was created to discuss specific aspects of frailty and/ or sarcopenia stemming from the most recent (often still unpublished research) with the final aim of providing updated recommendations. The ICFSR Task Force includes an international panel of Key Opinion Leaders in clinical and basic research, representatives from the pharmaceutical and nutritional industries, and members of non-profit organizations. As it has regularly occurred in previous ICFSR meetings, the Task Force gathered on September 28, 2021, to focus on the specific challenges of developing drugs for frailty and sarcopenia. The present report summarizes the presentations and discussions.

## Frailty and sarcopenia trials: the SPRINTT project experience

Conventional medicine is often inadequate for managing the complex medical needs of frail older people, frequently resulting in misdiagnosis and mistreatment. Furthermore, the biomedical model of disease becomes progressively less clinically relevant as people age, whereas his/her functions and reserves assume greater importance in ensuring healthy aging. In this context, special interest has been devoted over the past years to the sarcopenia and frailty conditions, focused on the skeletal muscle health and the physical functioning of the individual, respectively. Sarcopenia and frailty often coexist and overlap in the common feature of physical impairment (5). Sarcopenia has been frequently recognized as one of the strongest determinants of the frailty condition, a biological background affecting the person’s functional status. Consistently, randomized clinical trials (RCTs) testing the effects of lifestyle modifications (mainly physical activity and nutritional programs) have been conducted with the final aim of reducing the onset of disability in older people (7). In this context, the most relevant example is probably the Lifestyle Interventions and Independence for Elders (LIFE) study (8), a multicenter RCT enrolling about 1,600 sedentary people aged 70 and older with physical impairment (i.e., a Short Physical Performance Battery [SPPB] score of 9 or below) in the absence of mobility disability (i.e., able to walk 400 meters). Participants were randomized to a physical activity intervention versus a health education program. Results demonstrated that a moderate-intensity physical activity program can reduce the incidence of major mobility disability. Furthermore, the study supported the importance of the lifestyle in defining the risk profile, the reversibility of the disabling process, and the possibility of conducting physical activity even at very old age.

Following on that experience, the Innovative Medicines Initiative (IMI) funded the “Sarcopenia and Physical Frailty in Older People: Multi-component Treatment Strategies” (SPRINTT) project (9), with the following objectives:

1. To provide an operationalization of physical frailty and sarcopenia (PF&S) as an objectively measurable condition;
2. To identify a specific target population of older adults at risk of adverse outcomes with unmet medical needs;
3. To evaluate the effectiveness of a multicomponent intervention in the prevention of mobility disability;
4. To identify and validate biomarkers (for risk stratification, diagnostic, prognostic, and follow-up purposes) for the condition of PF&S.

The SPRINTT consortium was initially focused during the first months of activity at theoretically framing the perimeter of PF&S, paying special attention to the requirements of the regulatory agencies for the approval of novel clinical conditions (10). In fact, in this conceptual model, it was assumed that all the factors defining PF&S should be measurable (11). Moreover, low muscle mass (assessed by dual energy X-ray absorptiometry [DXA]) was identified as the biological substrate of the PF&S condition. The definition of the thresholds indicating the presence of low appendicular lean mass was based on the criteria proposed by the Foundation for the National Institutes of Health (FNIH) Sarcopenia I Project (12). Finally, the clinical phenotype of the PF&S was characterized by the presence of poor balance, slow gait speed, and/or muscle weakness, thus reflecting a physical impairment that is measurable through the SPPB.

The SPRINTT study included 1,566 people aged 70 years and older, across 16 sites in 11 European countries. The main eligibility criteria were an SPPB score between 3 and 9, the ability to complete the 400-m walk test within 15 minutes, and the presence of low appendicular lean mass.

Participants were randomized to a multi-component intervention (based on structured physical activity, nutritional assessment/counseling, and implementation of Information & Communication Technology [ICT] solutions) versus a healthy aging lifestyle education program. The primary outcome of interest for the SPRINTT trial was the incident mobility disability, defined as the onset of inability to complete the 400-m walk test (13). The main characteristics of the SPRINTT participants are presented in Table 1. As evident, the population is quite old, with a mean age of almost 79 years, and prevalently composed of women (more than 70%). Interestingly, although sarcopenia is a crucial component of the PF&S condition, and one might think of a relatively cachectic population, the mean body mass index describes the sample as clearly overweight. In other words, PF&S might be hidden behind the so-called sarcopenic obesity. Furthermore, consistently with the eligibility criteria, participants were mostly independent in both the basic and instrumental activities of daily living but presented a clear physical impairment as expressed by the low SPPB value.

**Table 1:** Demographics of the SPRINTT trial participants

**Characteristics**	**(n=1566)**
Age (years), mean α SD	78.9 α 5.8
Gender (female), n (%)	1119 (71.5)
Race/ethnicity, n (%)	
White	1380 (88.1)
Asian	17 (1.1)
African American/black	2 (0.1)
Other	4 (0.3)
Refused/missing	163 (10.4)
BMI (kg/m^2^), mean α SD	28.6 α 6.0
Calf circumference (cm), mean α SD	35.0 α 4.4
ADL score, mean α SD	5.6 α 0.6
IADL score, mean α SD	7.3 α 1.2
SARC-F score, mean α SD	2.9 α 1.9
MMSE score, mean α SD	27.9 α 1.8
SPPB summary score, mean α SD	6.7 α 1.4
4-m walk speed (m/s), mean α SD	0.73 α 0.19
Time to walk 400 m (min), mean α SD	8.69 α 2.45
400-m walk speed (m/s), mean α SD	0.82 α 0.21
ALM (kg), mean α SD	
Men	21.13 α 3.52
Women	14.73 α 2.15
ALM_BMI_, mean α SD	
Men	0.725 α 0.083
Women	0.529 α 0.076
Any cardiovascular medical history, n (%)	1109 (70.8)
Chronic lung disease, n (%)	242 (15.5)
Stroke or brain hemorrhage, n (%)	106 (6.8)
Cancer (excluding minor skin cancer), n (%)	217 (13.9)
Diabetes mellitus, n (%)	330 (21.1)
Osteoarthritis, n (%)	1204 (76.9)
Falls(s) in past year, n (%)	694 (44.3)
Injurious fall(s) in past year, n (%)	233 (14.9)
Previous hip fracture(s), n (%)	94 (6.0)
Previous non-femoral fracture(s), n (%)	505 (32.2)

ADL, activities of daily living; aLM, appendicular lean mass; ALM_BMI_, appendicular lean mass to body mass index ratio; BMI, body mass index; IADL, instrumental activities of daily living; MMSE, Mini Mental State Examination; SPPB, short physical performance battery, Adapted from (44) under the Creative Commons CC-BY-NC-ND license

The SPRINTT project was completed at the end of 2020 and the results (including those of the trial) have been finalized and will soon be published. An example of design of a randomized controlled trial on physical frailty and sarcopenia stemming from the SPRINTT experience is provided in Table 2.

**Table 2:** Example and suggestions for the design of randomized controlled trial on frailty and sarcopenia.

Primary outcome	Incident mobility disability. The 400-meter walk test represents a validated measure for defining this early step of the disabling cascade.
Secondary outcome	Clinically relevant modifications in muscle strength and/or muscle function. See the studies showing the changes of physical performance (i.e., Short Physical Performance Battery, gait speed) and muscle strength (i.e., handgrip strength) measures that define the minimal or significant clinical relevance. Body composition modifications (i.e., appendicular lean mass [adjusted or not by adiposity], overall body muscle mass) Falls Use of health and social care services (e.g., Emergency Room admissions, hospitalizations, fall-related hospitalization, length of hospital stay, institutionalization, outpatient visits…) Adverse Drug Reactions Mortality
Eligibility criteria to consider	A measure of the individual’s risk profile (e.g., frailty) Life threatening conditions (i.e., less than 6 months of life expectancy) Capacity to adhere to the study protocol. This should, however, be read as the need of being as much inclusive as possible, adapting the design to the complexity of the older person
Eligibility criteria to not consider	Age. The inclusion of a chronological age criterion may introduce ageism. Furthermore, chronological age is not equivalent to biological age. Consider instead a measure of frailty to biologically stratify the risk profile
Follow-up length	Depending on the primary outcome, the risk profile of participants, and the sample size. At least 6 months for obtaining hard outcomes, such as mobility disability

## Myostatin monoclonal antibodies

The discovery of growth/differentiation factor-8 (GDF-8), also known as myostatin, as an inhibitor of skeletal muscle growth, has led to high expectations in the scientific community. The mutation of the myostatin gene in mice was initially associated with a substantial increase in muscle mass (14). Subsequently, it has been observed that the larger size was also characterized by a higher number of muscle fibers. In 2004, Schuelke et al. (15) reported the case of a child who, due to a mutation in the myostatin gene, exhibited greater muscle mass and unusual strength compared to peers. Also, several members of his family showed a higher-than-usual strength. These findings led to hypothesize possible applications of myostatin inhibition as a therapeutic target in patients with muscle-wasting conditions (16).

**Figure 1:**
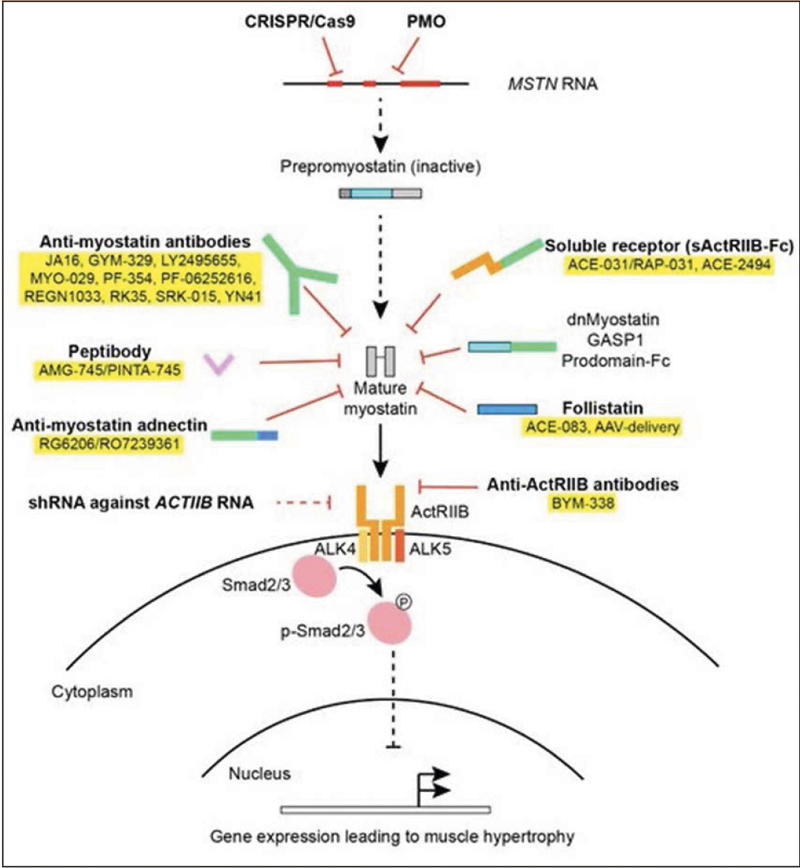
Overview of treatment approaches used in myostatin inhibition

Since then, various approaches have been used in this field (17). In particular, 1) the systemic administration of antibodies against myostatin, 2) the liver-mediated overexpression of a soluble receptor of the activin type IIB (sActRIIB), and 3) the administration of antibodies against the myostatin receptor, activin-receptor type II (ActRII) have been considered (Figure 1).

The first lesson from the myostatin inhibitors comes from the animal models. In 2002, Bogdanovic et al. (18) showed that the block of myostatin is associated with an increase in muscle mass and strength and a parallel decrease of muscle damage in mice. The authors suggested that the intervention could be particularly beneficial for muscular dystrophies. However, when the first clinical trial of anti-myostatin agents (19) was conducted on three forms of dystrophies (i.e., Becker muscular dystrophy, facioscapulohumeral dystrophy, and limb-girdle muscular dystrophy), only a marginal increase in muscle mass and no effects on strength and function were reported. However, it should be noted that the study was not adequately powered to evaluate the efficacy of the intervention.

Subsequently, Attie et al. (20) reported that a single dose of ACE-031 (i.e., a soluble form of the ActRIIB receptor) increases lean body mass (LBM; i.e., about one kg in four weeks) in healthy postmenopausal women. In 2015, a phase II study testing a single dose of a myostatin antibody (i.e., Landogrozumab) in a population of older adults reporting recent history of falls showed an increase of LBM (about 0.7 kg at 24 weeks). The first study recruiting older adults with sarcopenia (21) tested a single dose of Bimagrumab, a human monoclonal antibody that inhibits the binding of multiple ligands (i.e., myostatin, activin A) by acting on the ActRII. Results showed an increase in LBM (i.e., 1.6 kg at four weeks and 2.0 kg at 16 weeks), muscle strength, and function. Therefore, it could be suggested that blocking multiple ligands may represent a more effective strategy for promoting muscle hypertrophy compared to acting on a single/specific mechanism, as the myostatin alone. Recently, Rooks et al. (22) tested the efficacy of Bimagrumab in 180 older adults with sarcopenia. The positive effects on the LBM were confirmed (i.e., 1.93 kg at 12 weeks and 2.02 kg at 24 weeks). While, no significant improvements were observed for measures of muscle strength and function, the subgroup of subjects with slower walking speed at baseline showed potential responsiveness via statistically significant and clinically meaningful improvements in gait speed and 6-minute walk test (6MWT) distance versus placebo at Week 16 of the 24-week trial.

The inhibition of myostatin through the above-mentioned mechanisms has been described as generally safe and tolerable. Commonly reported adverse events tend to include muscle symptoms (i.e., cramp, twitch), gastrointestinal symptoms (i.e., diarrhea, nausea), acne/rash, local skin reactions to subcutaneous injection administration, transient increase of pancreatic enzymes, fatigue, and myalgia.

It should not be overlooked the role played by the age-related body composition changes, responsible for an increase in visceral abdominal fat and fat infiltration of the muscle, leading to the induction of myocytic apoptosis (23–25). Intramuscular fat infiltration can exert its detrimental effects on muscle strength and quality, negatively affecting mobility function (26–28). Recently, it has been reported that blocking the ActRII with Bimagrumab in overweight or obese patients with type 2 diabetes leads to a parallel decrease in fat mass (29), and in accord with the sarcopenia RCT results described above.

In summary, findings from animal models do not always translate to humans, and early proof-of-concept studies do not always replicate in larger-scale studies. A direct action promoting the muscle mass increase does not necessarily translate into a parallel improvement of strength or function. Patient heterogeneity and comorbidities likely present confounding issues in this regards that may impose limited response potential to PF&S therapies. Thus, discerning which groups of patients that may be responders will be essential to advancing therapeutic candidates; and just as importantly, understanding why others may be poor-responders. Indeed, these lessons will shape future generations of drugs developed to prevent or reverse muscle wasting, which still represents an unmet clinical need.

## Novel pharmacological approaches

The development of novel therapeutic strategies against frailty and sarcopenia is highly active. Of note, some of these approaches include drugs commonly prescribed for other conditions. Table 3 presents an overview of the ongoing clinical trials currently evaluating drugs against sarcopenia. A promising example is represented by the orally administered agent called Sarconeos (BIO101), produced by Biophytis (Paris, France). Sarconeos is based on a 95% 20-hydroxyecdysone extract purified from a plant. More in details, Sarcopenos is a Mas receptor activator in the renin-angiotensin system (30). In myocytes, it acts as trigger of the AKT and AMPK pathways, which results in increased protein synthesis and energy production, respectively. Sarconeos is currently being tested in the SARcopenia and sarcopenic obesity in patients Aged ≥ 65 years (SARA) program. Nested in the SARA program, the SARA-INT (ClinicalTrials.gov NCT03452488) is a phase II trial aimed at evaluating the safety of Sarconeosand its efficacy on mobility after a 6-months administration to sarcopenic patients. The main eligibility criteria are: age ≥ 65 years; SPPB score ≤8; low muscle mass according to the Foundation for the National Institutes of Health (FNIH) criteria for sarcopenia; ability to perform the 400-m walk test within 15 minutes. The primary endpoint of the SARA-INT study is the change in gait speed (measured during the 400-m walk test). A total of 232 subjects (Full Analysis Set/FAS) have been included in the study, and two dosages of Sarconeos (i.e., 175 mg twice a day and 350 mg twice a day for six months) have been tested and compared to placebo. The preliminary results of the trial have been anticipated during the Task Force meeting and discussed among the participants. In the full analysis set, the Sarconeos 350 mg bid treatment showed an improvement in the 400-m walk test compared to placebo after 6 months of treatment, although statistical significance was not reached. A statistically significant benefit, close to the minimal clinically relevant difference (31), was reported in the per-protocol analysis. The positive effect was confirmed in PP subgroup analyses, showing gait speed improvements at the 400-m walk test in slow walkers (i.e., <0.8 at the 400-m walk test), obese subjects (i.e., percentage of body fat >25% and >35% in men and women, respectively) and subjects with a chair stand sub-score ≤2 at the SPPB (32). Unfortunately, due to the COVID-19 pandemic, the end-of-treatment assessments are missing for approximately half of the participants (i.e., 55% of the FAS dataset), potentially impacting on the study findings.

**Table:** 

**Drug intervention**	**Mechanism of action**	**NCT number**	**Phase**
Androgel (Testosterone Gel), Anastrozole	Testosterone replacement, Aromatase inhibition	NCT00104572	Phase 2
LPCN 1148	Testosterone replacement	NCT04874350	Phase 2
Levothyroxin	Thyroid hormone replacement	NCT04354896	Phase 4
Anamorelin Hydrochloride	Ghrelin receptor agonist	NCT04021706	Phase 1
Testosterone	Testosterone replacement	NCT03995251	Not applicable
Renamezin	Mitochondrial function	NCT03788252	Phase 4
Melatonin	Melatonin hormone replacement	NCT03784495	Not applicable
BIO101 (20-hydroxyecdysone)	MAS receptor activation	NCT03452488	Phase 2
Oxytocin	Oxytocin hormone replacement	NCT03119610	Phase 1 and 2
Sustanon 250, Zoladex	estosterone replacement, Gonadotropin replacement	NCT03054168	Phase 3
Testosterone	Testosterone replacement	NCT02938923	Phase 3
Valsartan	Angiotensin II receptor blockade	NCT02606279	Not Applicable
Vitamin D3	Vitamin D replacement	NCT02594579	Phase 3
Cetylpyridinium Chloride	Antiseptic activity	NCT02594579	Phase 3
Bimagrumab	Activin receptor inhibition	NCT02468674	Phase 2
GLP-1, Insulin Actrapid, GIP	Hormone replacement	NCT02370745	Not Applicable
Bimagrumab	Activin receptor inhibition	NCT02333331	Phase 2
Alfacalcidol	Vitamin D replacement	NCT02327091	Phase 3
Pioglitazone, Insulin, Octreotide	Insulin sensitization, PPARγ activation, somatostatin agonist	NCT02305069	Not Applicable
Cetylpyridinium chloride	Antimicrobial activity	NCT02297997	Early Phase 1
Losartan	Angiotensin receptor blockade	NCT01989793	Phase 2
REGN1033 (Trevogrumab)	Myostatin or activin inhibition	NCT01963598	Phase 2
Ghrelin	Ghrelin replacement therapy	NCT01898611	Phase 2
Ibuprofen	Non-selective, reversible inhibition of COX-1 and COX-2	NCT01886196	Not Applicable
Metformin	Inhibition of hepatic gluconeogenesis	NCT01804049	Phase 1 and 2
Vitamin D	Vitamin D replacement	NCT01666522	Not Applicable
Allopurinol	Xanthine oxidase inhibition	NCT01550107	Phase 4
Testosterone enanthate	Testosterone replacement	NCT01417364	Phase 4
Vitamin D3	Vitamin D replacement	NCT00986596	Not Applicable
Testosterone	Testosterone replacement	NCT00957801	Phase 4
Rapamycin, Sodium nitroprusside	Inhibition of mTOR, vasodilatation	NCT00891696	Phase 1
Insulin, L-NMMA, Sodium Nitroprusside	Insulin replacement, NOS inhibition, Vasodilatation	NCT00690534	Phase 1
MK-0773	Selective androgen receptor modulation	NCT00529659	Phase 2
Testosterone Enanthate, Finasteride	Testosterone replacement, 5-alpha reductase inhibition	NCT00475501	Phase 2
MK-677	Selective androgen receptor modulation	NCT00474279	Phase 1 and 2
Pioglitazone	Insulin sensitization, PPARγ activation	NCT00315146	Phase 4
Androgen replacement	Androgen replacement	NCT00254371	Not Applicable
Topical testosterone gel 1%	Testosterone replacement	NCT00240981	Phase 4
Dehydroepiandrosterone	Dehydroepiandrosterone replacement	NCT00205686	Phase 3
Transdermal testosterone gel	Testosterone replacement	NCT00190060	Phase 4
Topical testosterone	Testosterone replacement	NCT00183040	Phase 2
MK0677	Selective androgen receptor modulation	NCT00128115	Phase 2

GIP: Glucose-dependent insulinotropic polypeptid; GLP-1: Glucagon-like peptide 1; mTOR: mammalian target of rapamycin; NOS: Nitric oxide synthase; PPARγ: Peroxisome proliferator-activated receptor gamma

Among the ongoing studies testing interventions against frailty, a phase IIB RCT (ClinicalTrials.gov NCT03169231) exploring the effects of Lomecel-B, an allogeneic bone marrow-derived product featuring mesenchymal signaling cells (MSCs) formulation, was presented (32). The major eligibility criteria included: age ≥70 years; a Clinical Frailty Scale (CFS) (33) score of 5 or 6; a 6MWT distance of 200 to 400 meters; and concentration of serum tumor necrosis factor-alpha (TNF-α) equal to or higher than 2.5 pg/mL as a measure of inflammaging. A total of 148 subjects (135 mildly frail and 13 moderately frail according to the CFS) were recruited and randomized to receive a single peripheral intravenous infusion of Lomecel-B at one of four dosages (25, 50, 100, or 200 million cells), or placebo. The primary endpoint was the change in the 6MWT distance at 180 days post-treatment. Preliminary results were presented for the first time. Briefly, the results indicated a dose-respons relationship of Lomecel-B to increased 6MWT distance, with statistically significant increase in 6MWT in the highest 3 Lomecel-B dosages (i.e., 50 million, 100 million and 200 million cells). No significant changes in 6MWT were found in the placebo or lowest dose of Lomecel-B group. Notably, the 6MWT distance increased about 50 meters in the 200 million dose group by day 180, and was sustained through day 270 and highly significantly different from the change in placebo (p=0.007) (34). These 6MWT increases exceeded published estimates of minimal clinically important differences for frail and older patients of about 20 meters (35, 36).

## Clinical trials on sarcopenia and frailty during the COVID-19 pandemic

The COVID-19 pandemic has drastically modified the clinical routine and introduced significant challenges in research (37). During the first phase of the pandemic, there was a nearly complete shutdown worldwide of the non-COVID research to limit the spread of the virus and adhere to social restrictions applied by the different governments. It became immediately evident that the design of research activities as planned before the COVID-19 pandemic was substantially inadequate and inapplicable in most cases.

The COVID-19 pandemic imposed significant impediments in the enrollment of clinical trial participants. It was not uncommon for studies to have been stopped, or some new initiatives upheld/suspended by either the sponsors or the regional lockdowns (38). It was frequently necessary to adapt the study methods to the new and evolving scenario. Diverse strategies to maximize the safety of the study participants as well as the study staff and to minimize the spread of the virus have been proposed and implemented to maintain the studies running safely or restore the research activities [39,40]. Consequently, the required modifications have contributed to delays and extra costs. Frequently, on-site and in-person assessments were converted into virtual contacts or at-home visits; investigational products had to be delivered at study subject residence; and questionnaires have been administered remotely (38). Frequently, the study protocols were streamlined to allow greater scheduling flexibility and to focus on rank-order collecting data of primary/essential endpoints. Occasionally, more flexible consent procedures, both paper-based or electronic, were implemented in some study sites to facilitate enrollment.

Physical function measures are frequently used as primary and secondary outcomes in the assessment of older people living with frailty and sarcopenia in both research and clinical practice (40–42). The participants and study staff could be at risk of getting infected in the exercise lab environment, and is of excepetional concern for the PF&S studies, whose study subjects are highly axposed to severe outcomes from the SARS-CoV-2 infection. The lab policies had to comply with varying institutional, state, and federal policies.

Storer et al. (40) have published guidance on steps that exercise laboratories can implement to minimize infection risk during the performance of laboratory-based tests of physical function and muscle performance during epidemics of this and other respiratory infections. For risk assessment, Storer et al. (40) categorize the procedures for the assessment of physical function and muscle performance into two broad categories:

- Category 1: procedures that are not aerosol-generating, and result in only a marginal increase in minute ventilation (i.e., SPPB, short distance walks, timed get-up and go, stair climbing, grip strength). These types of tests are associated with only a minimal increase in the risk of infection;
- Category 2: tests that are aerosol-generating and are associated with a marked increase in minute ventilation (e.g., the cardiopulmonary exercise test and the 6MWT in some frail older people).

Storer et al. propose a steped approach to maximize safety during the physical function assessments. The first step is aimed at evaluating the benefit to risk ratio of the study procedures. Accordingly, if assessments are included as exploratory outcomes, it may be prudent to consider postponing or even eliminating them. On the other hand, if the assessments are identified as primary or important secondary outcomes, it should be determined whether the test belongs in Category 1 or 2. Safety procedures for Category 1 tests include the optimal use of personal protective equipment, frequent sanitization of surfaces, decluttering of the lab, and maintenance of sufficient distancing (i.e., at least 6 feet). For Category 2 procedures, in addition to all the Category 1 preventive strategies, the optimization of ventilation in the laboratory and the use of HEPA filters may be needed.

As mentioned above, the COVID-19 pandemic incentivized the application of remote sensing technologies for some outcome assessments and study procedures. However, it should be noted that the results of the remote assessments may be quite different from the standard ones performed in a clinical research unit. In a recent study, Hale et al. (43) measured and compared measures of gait speed in the home measured using an accelerometer with the lab-based measurement of gait speed in a 6MWT in HIV-positive and HIV-negative men. They did not find substantial differences in the lab-based 6MWT gait speed between HIV-positive and HIV-negative men, whereas the accelerometer-derived gait speed in the home setting was significantly lower than that measured in the exercise laboratory in HIV-positive participants. These data show that the variables generated from home-based and lab-based assessments should not be used interchangeably because these assessments in the home setting and the laboratory setting potentially differ in their construct and characteristics. Today, many trials are collecting data remotely for activity measures using wearable sensors (in particular, actimeters). These wearable remote sensing tools may enable real-life assessment of many important aspects of physical performance beyond what traditional laboratory-based tests may do. These promising home-based assessments of physical function using remote sensing technologies need further standardization and validation. . There is indeed the need to better validate these novel measures and to generate normative data for home-based remotely collected physical function assessments.

The COVID-19 pandemic has reshaped how clinical trials were conducted, also requiring the implementation of procedures to enable the remote administration of visits and tests. To support the validity of these adaptations, the standardization of the remote procedures (also those focused on the physical function assessment) is needed. When assessing study participants, the direct and indirect impacts of the pandemic should be considered for potential changes on their biological, clinical, and social characteristics.

## Conclusions

Over the last decades, increased research efforts have been devoted to frailty and sarcopenia. This has helped pave the way for clinical exploration of pharmacological and lifestyle interventions, and several promising agents are in the pipeline and currently being explored. It is agreed that measures of physical function are critical in this context. Nutritional and exercise interventions remain the first-line approach in the management of older people with frailty and sarcopenia. However, new drug candidates may find an ideal positioning, particularly among non-responders to lifestyle modifications or those not able to participate because of biological, clinical, and/or social factors. Finally, the importance of combining pharmaceutical and exercise interventions should not be overlooked in its potential. The simultaneous adoption of independent strategies targeting the quantitative and qualitative dimensions of the sarcopenia phenomenon may generate beneficial synergies. This approach may even become a requirement for proving the effectiveness of the pharmaceutical intervention.
